# A Compact and Flexible UHF RFID Tag Antenna for Massive IoT Devices in 5G System

**DOI:** 10.3390/s20195713

**Published:** 2020-10-08

**Authors:** Muhammad Hussain, Yasar Amin, Kyung-Geun Lee

**Affiliations:** 1 Network Research Lab (NRL), Information and Communication Engineering Department, Sejong University, Seoul 05006, Korea; engrhussain2010@sju.ac.kr; 2 ACTSENA Research Group, Department of Telecommunication Engineering, University of Engineering and Technology, Taxila, Punjab 47050, Pakistan; yasar.amin@uettaxila.edu.pk

**Keywords:** IoT, I-RFID, plausible read range, meandering angle technique (MAT), passive UHF tag, smart cities, 5G systems

## Abstract

Upcoming 5th-generation (5G) systems incorporate physical objects (referred to as things), which sense the presence of components such as gears, gadgets, and sensors. They may transmit many kinds of states in the smart city context, such as new deals at malls, safe distances on roads, patient heart rhythms (especially in hospitals), and logistic control at aerodromes and seaports around the world. These serve to form the so-called future internet of things (IoT). From this futuristic perspective, everything should have its own identity. In this context, radio frequency identification (RFID) plays a specific role, which provides wireless communications in a secure manner. Passive RFID tags carry out work using the energy harvested among massive systems. RFID has been habitually realized as a prerequisite for IoT, the combination of which is called IoT RFID (I-RFID). For the current scenario, such tags should be productive, low-profile, compact, easily mountable, and have eco-friendly features. The presently available tags are not cost-effective and have not been proven as green tags for environmentally friendly IoT in 5G systems nor are they suitable for long-range communications in 5G systems. The proposed I-RFID tag uses the meandering angle technique (MAT) to construct a design that satisfies the features of a lower-cost printed antenna over the worldwide UHF RFID band standard (860–960 MHz). In our research, tag MAT antennas are fabricated on paper-based Korsnäs by screen- and flexo-printing, which have lowest simulated effective outcomes with dielectric variation due to humidity and have a plausible read range (RR) for European (EU; 866–868 MHz) and North American (NA; 902–928 MHz) UHF band standards. The I-RFID tag size is reduced by 36% to 38% w.r.t. a previously published case, the tag gain has been improved by 23.6% to 33.12%, and its read range has been enhanced by 50.9% and 59.6% for EU and NA UHF bands, respectively. It provides impressive performance on some platforms (e.g., plastic, paper, and glass), thereby providing a new state-of-the-art I-RFID tag with better qualities in 5G systems.

## 1. Introduction

**Figure 1:**
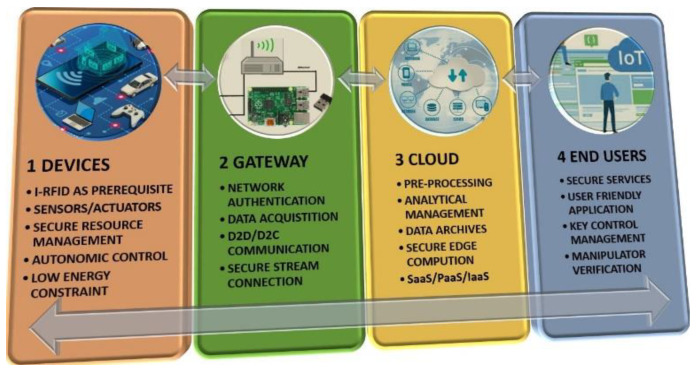
Internet of things (IoT) four-tier architecture for smart X environments. Each tier is linked with each other through internet gateway access. Under this scenario, end users can easily manage and control the devices tier.

The world market is expected to become saturated with new-tech internet of things (IoT) massive devices as a part of the upcoming 5th-generation (5G) systems, such as gears, gadgets, sensors, and actuators. In the futuristic IoT perspective, all physical objects/devices (referred to as things) can sense surrounding environments and transmit their current status. They assert their existence among the IoT surroundings, which means that they must synchronously update their data tables in the IoT cloud. For this scenario, the IoT four-tier architecture is depicted in Figure 1.

The device tier consists of sensors, actuators, and IoT radio frequency identification (I-RFID) tags for data collection under secure autonomic resource management control, where I-RFID has been consistently appreciated as a prerequisite for IoT [1]. The gateway tier provides secure and authenticated data stream connections, utilizing both device-to-device (D2D) and device-to-cloud (D2C) communication. The cloud tier systematically deals with the informative data through pre-processing, data archival, and edge computing. Satisfactory applications for authenticated user control and management are executed by clients in the end user tier.

**Figure 2:**
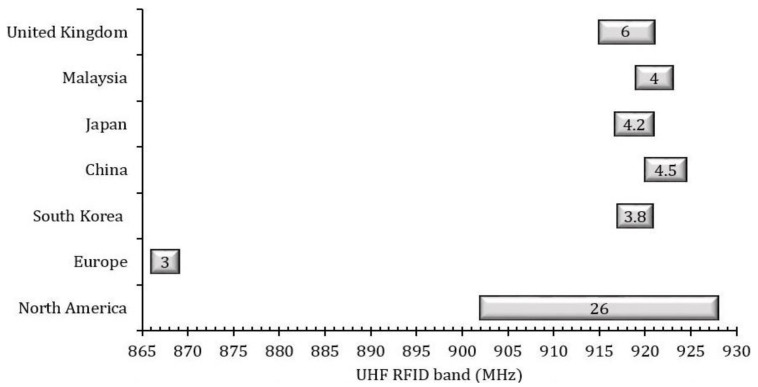
Ultra-high frequency (UHF) radio frequency identification (RFID) band (865–930 MHz) standards of different countries.

In the modern technological era, I-RFID technologies assume an imperative role in the challenging IoT environments of smart cities, such as securing massive logistics control at seaports and aerodromes, enabling smart retailing at marts, allowing for quick immigration clearance at airports, and facilitating e-health and waste management. RFID automation utilizes relevant frequency band standards, such as low frequency (LF): 125–134.2 KHz, high frequency (HF): 13.56 MHz, ultra-high frequency (UHF): 860–960 MHz, and super-high frequency (SHF) 2.45 GHz. Numerous countries have distinct effective isotropic radiated power (EIRP) constraints for UHF RFID tags; for example, North America, South Korea, and Japan have 4 W EIRP; Europe, China, and Malaysia have 3.28 W; and the EIRP of the United Kingdom is 6.56 W. The UHF bandwidth credentials of each country are shown in Figure 2. I-RFID automation has many potential applications, such as automobile identification [2], e-ticketing, e-tolling, logistics tracking [3,4,5], road traffic congestion and smart city surveillance [6], hospitals [7], schools (e.g., teaching management systems) [8], highways (IoT networks) [9], airports [10], and in textile industries [11,12]. These applications require low-cost and efficient I-RFID tags with simple mountable features that suit many different things. These tags consist of an antenna and microchip. A tag’s circuitry operation acts by harvesting RF energy that is emitted by a reader or interrogator (in the form of electromagnetic waves) and converting it into direct current (DC) power to trigger the integrated circuit (IC) by rectification and the envelope detection method. Then, the IC’s encrypted data are modulated with the reader carrier wave, and the resultant signal is sent back to the reader by a back-scattering process with the same antenna that was first used to receive the harvested energy.

Tags are arranged, depending on the power source used, in three categories: (a) Passive/Inactive tags, (b) Semi-Active tags, and (c) Active tags. Inactive tags are normally utilized due to their simplicity and low industrial price. I-RFID is a secure real-time processing mechanism in which transmission arises between the RFID tag and the interrogating reader, which continuously synchronizes and collaborates with the servers to update the relevant data tables [3].

The tag accepts the harvested RF power and directs the data to the interrogator using two communication approaches: (a) Near-field communication (NFC) by inductive coupling or (b) far-field communication (FFC) by radar/back-scattering. The information contains data relating to finding the object/thing, which is stored in the flip-chip package [13]. For tag activation, the harvested RF power originating from RFID reader ought to be sufficient, as opposed to the threshold power of the IC after crossing over the barrier losses [13,14,15].

A transformative approach has been presented, in [16], to accomplish robust versatile RFID antennas on flexible paper substrates for roll-to-roll production lines in green electronics. Thinned tags depict typical outputs for numerous challenges of antennas, in terms of ruggedness, trustworthiness, and flexing performance. Tag performance has been scrutinized after 50 and 100 bends after fabrication by flexo printing. The impedance of the tags was not affected too much after the bending process. For this reason, these tags were chosen for this study, due to their marvelous characteristics for item-level tracking [16]. 

### Related Research Work

In [17], Monza-R6 was utilized for RFID tags for bank card and person tracking with three operations enabled: RFID, NFC, and Europay, Mastercard, and Visa (EMV) chip. For high gain, an aluminum patch has been made by radiation of a polyethylene terephthalate (PET) substrate (*ε_r_* = 3) for the EU UHF RFID band; however, its development and testing phases have not been reported. A cavity structure tag antenna (CSTA) and a bottom metal tag antenna (BMTA) were structured for metal body logistics monitoring using Higgs-3 flip-chip package. The directivity of the CSTA was more noteworthy than that of the BMTA [18]; however, its figure of merit (FOM) was low on the basis of the read range to antenna size ratio (as discussed in [19]).

For a body area network (BAN), a tag meandered antenna was manufactured using aluminum and polycarbonate (*ε_r_* = 3.9). Polydimethylsiloxane (PDMS) material (10 mm thickness) was spread as a layer, in order to eliminate the body effect [20]. However, the size and the cost of the tag were higher, making it unsuitable for low-profile tagging. A multi-function RFID tag antenna has been presented for the UHF RFID standard. The inductive slotted-T technique was introduced for perfect conjugate matching with the MURATA (LXMS31ACNA-010) IC in single-layer tags. For both scenarios (i.e., Eu and NA UHF standard), read ranges were 5 m and 4 m in free-space communications, respectively [21]. However, the fact-finding scenario was not investigated with respect to impedance matching and transmitted power [21].

For metallic objects, the S11 parameter has been optimized through the electrical permittivity (*ε_r_*) effect from 4.3 to 4.7, while the bent stub method has been used for impedance matching with the MURATA (LXMS21ACMF-183) IC. Two parameters are highly important for device-to-device (D2D) communication: tag permittivity and the reader’s transmitted power. These directly affect the frequency shift and tag read range, correspondingly [22]. For high-temperature applications, a compact UHF RFID ceramic tag with quarter-mode patch antennas has been introduced. The tag placed-on-metal performance was tested with the heating process section Valp-ARE magnetic stirrer to temperatures above 100 °C. Under these temperature circumstances, the tag read range performance decreased gradually w.r.t. enhancement along with heating. However, the tag maximum read range was 1 m at room temperature, which is not appropriate for the considered system [23]. 

A 24 GHz MIMO multilayer Yagi antenna has been manufactured for 5G applications in IoT scenarios, which has 10.9 dBi gain and 6.9 GHz bandwidth. The planar structure equivalent had 8.9 dBi gain and 4.42 GHz bandwidth [24]. However, its large size is a drawback, which leads to high manufacturing costs [25]. An empty cavity tag (ECT) of size 140 mm × 60 mm × 10 mm has been produced for metallic (as well as non-metallic) applications. Using 3D printing, the plastic cavity is made of polylactic acid (PLA; *ε_r_* = 1.3) and acrylonitrile butadiene styrene co-polymer (ABS; *ε_r_* = 2.8) filaments. A copper radiator was used with a Higgs 4 (−20.5 dBm) chipset for perfect impedance matching. This ECT was designed under the North American (NA) UHF RFID standard. However, the tag performance was not investigated under differing humidity and temperature conditions [26]. 

In [16], the series and shunt stubs technique was utilized for perfect impedance matching using the NXP ucode G2XM flip-chip package. Meandered shaped antennas are directly printable on paper karsnos, Teonix Q51, and Kapton HN substrates with screen, flexo, and inkjet printing, respectively, for green electronic tags. In [16], high cogency concerning strain and di-electric atmosphere were confirmed. The authors aimed to pay additional attention to the reading range (RR), bandwidth (BW), and compactness of the green tag in future development. In this work, we propose meandering angle technique (MAT) optimization and perfect impedance matching. The main contributions of the proposed work are as follows:

- Meandering angle formulation [27] for the MAT process, in order to minimize the antenna size and enhance the realized gain of the UHF RFID tag;
- We use the flip-chip package NXP-G2XM [28] for perfect impedance matching using the series and shunt stub technique [29];
- The antenna’s inductive behavior is guaranteed through the introduction of capacitive end tip-loading [16];
- A dielectric effect is achieved w.r.t. the atmospheric humidity level;
- The read range of the tag w.r.t. the reader’s EIRP and reader power sensitivity [29] is ensured in the IoT environment under the global UHF RFID standard (860–960 MHz) [13];
- We carry out a performance comparison of the proposed tag with previously published tags (PPTs) [16,17,18,20,21,22,23];
- We detail the indoor and outdoor I-RFID working mechanisms with the UHF RFID integrated reader (SL130) [30] for IoT smart X environments; and
- We demonstrate the tags integrability with mounting platforms (plastic, paper, and glass) [31] to enable massive IoT devices in 5G systems.

The rest of the paper is organized as follows: Section 2 presents the I-RFID tag antenna design and simulation in detail. Section 3 discusses the experimental results of the presented research work thoroughly. Finally, Section 4 concludes the paper.

## 2. I-RFID Tag Antenna Design and Simulation

**Figure 3:**
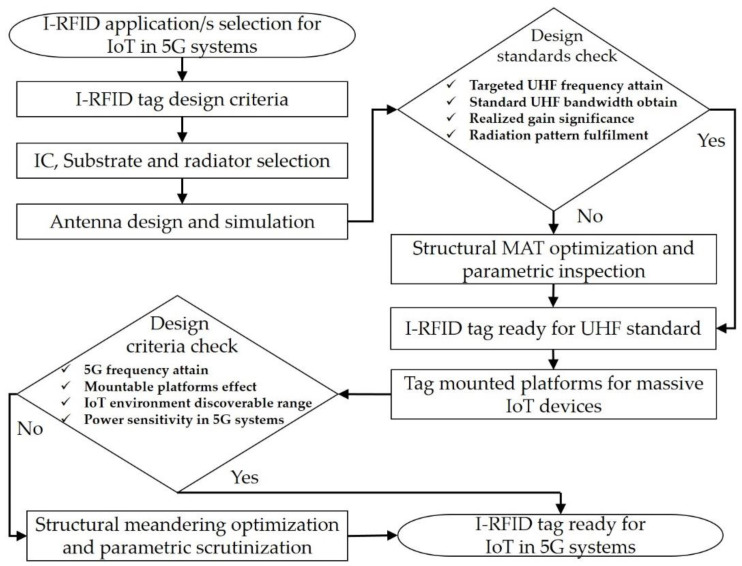
Flow chart of design process for I-RFID UHF tags for 5G IoT systems.

**Figure 4:**
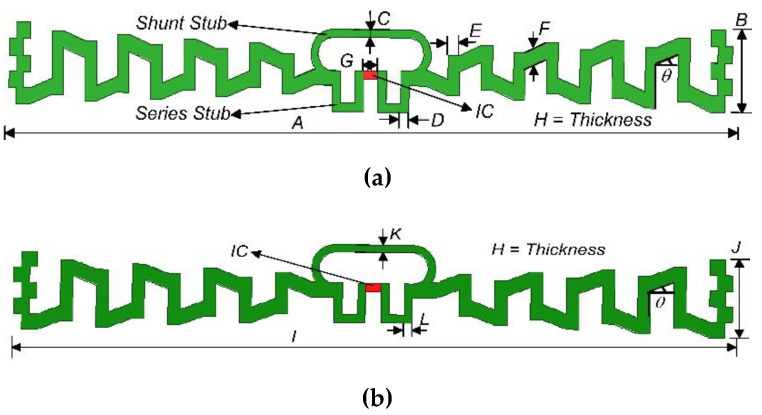
(**a**) Conductive Asahi paste I-RFID Tag for European UHF RFID standard and (**b**) conductive Asahi paste I-RFID Tag for North American UHF RFID standard.

The design procedure of the proposed I-RFID tag is demonstrated in Figure 3. MAT antennas are fabricated on Korsnäs substrate (375 µm) by flexo printing, with constraints of permittivity (*ε_r_*) = 3.3 and electrical tangent loss (*δ*) = 0.077 (at 25 °C). Asahi paste (LS-411AW) was used as an antenna radiator with a thickness of 15 µm. The tags were constructed using screen- and flexo-printing machineries. Its bulk conductivity (σ) 2–3 × 10^6^ S/m and its average conductivity value 2.5 × 10^6^ S/m were utilized in tag design. In this way, the total thickness of the designed tag was 390 µm (0.39 mm). Little power (−15 dBm) is needed to function the flip-chip (NXP-G2XM) [28]—at 915 MHz, its impedance is 22 − *j*195 Ω, and its geometric size is 3 mm × 3 mm, which was selected as the integrated circuit (IC) due to its passive UHF RFID MAT tag composition. The projected UHF I-RFID MAT tags are illustrated in Figure 4a for the European (EU) tag standard and Figure 4b for the North American (NA) tag standard, correspondingly.

The operational frequencies of the mutual UHF standards are 867 and 915 MHz for European (EU) and North America (NA), respectively [13,32]. From the bar chart of Figure 2, we can see that a European band antenna will also encompass the lower-frequency UHF band countries, while the North American tag will cover up the upper UHF band countries (e.g., South Korea, Japan, Malaysia, and the United Kingdom). The geometric boundaries of both MAT antennas are given in Table 1. Using the finite element method (FEM), simulations of the proposed project were accomplished using the ANSYS-HFSS™ software, with numerous methods (as detailed below).

### 2.1. Meandering of Dipole

The measurements of the dipole antenna were 346 and 328 mm for EU and NA UHF bands, respectively, which were formulated using the speed of light equation *c* = *fλ*, where *c* = 3 × 10^8^ m/s and *f* is the operating frequency (867 or 915 MHz) of the targeted UHF bands for Europe and North America, respectively. These are quite large and not appropriate for a small tag. For this reason, meandering of the dipole is accomplished to reduce the antenna’s length and allow for manufacturing small tags [13,33]. To make the antenna more inductive, the “*E*” length (depicted in Figure 4a) was introduced during meandering, as our flip-chip package NXP G2XM is more capacitive (22 − *j*195 Ω). Due to this, its length plays an important role in controlling the inductive behavior of the tag. The width “*E”* controls the capacitive behavior of the antenna. Two consecutive “*E”* lengths act as a capacitor but cancel their effect (due to the opposite field effect). For this reason, the meandering antennas demonstrate inductive behavior, instead of capacitive.

### 2.2. Meandering Angle Creation for Meandering Angle Technique (MAT)

Meandering angles were designed using Equations (1) and (2), which are tangent line–slope equations.

*y = kx + c* (1)

*tan θ* = (*y*2 − *y*1) / (*x*2 − *x*1). (2)

Equation (1) contains the slope *k* and the y-intercept *c*, thus forming a first-order pair; while second-order pairs of intercepted line values—*x*1, *x*2 and *y*1, *y*2—and the meandering angle *θ* in MAT form Equation (2) [27]. The MAT meandering elevation angles of *θ* = 0°, 15°, 30°, and 45° project onto the y-axis with the variation of x-axis values. At a plane angle (*θ* = 0°), the dimension of the antenna is 101.2 mm × 12.2 mm. With the angle variations at *θ* = 0°, 15°, 30°, and 45°, the tag antenna’s width fluctuates but the length is kept the same, as shown in Table 2. The most compact dimension (of 101.2 mm × 10.5 mm) was attained at *θ* = 30°. Its width did not reduce any more with increasing the angle. Its size does not remain more compact at *θ* = 45°, bandwidth and gain values decrease from 37 to 33 MHz and 2.75 to 1.75 dBi, respectively. This implies that the antenna tag was minimized at *θ* = 30°, attaining its maximum 37 MHz bandwidth (BW) and 2.75 dBi gain value. Similarly, the North American tag had minimum dimension at the angle *θ* = 30°, which was 92.4 mm × 10 mm, as elaborated in Table 2.

### 2.3. Impedance Matching Network

To find an ideal match between the radiating antenna and flip-chip, a conjugated matching network (CMN) was constructed. A matching network in comprised of series (open) and shunt (close) stubs. For a perfect impedance matching network (PIMN), both stubs are essential in the antenna scheme. This forms the double stub impedance matching technique (IMT). The loops technique [34] and the feeding loop technique [35] have been used for impedance network matching. The IC NXP-G2XM [28] is joined along the series (open) stubs of the matched network. Its size is 3 mm × 3 mm with eight leads. The first lead is soldered with the grounded part of the antenna, and the last lead is soldered with the ungrounded part of the antenna (whenever all other leads are free) [28]. The real part (R) of the impedance (Z = R + *j*X Ω) is controlled by the series stubs, and the shunt stubs fluctuate until the reactance (X) of the antenna becomes equal in magnitude but opposite in phase to the reactance of the flip-chip [29,33]. If Z_IC_ = 22 − *j*195 Ω is the flip-chip IC impedance, the point Z_A_ = 22 + *j*195 Ω would be the radiating antenna impedance, where the reactance of the tag is equal in magnitude but conflicting in phase. Along these lines, the conjugate impedance of the tag is perfectly matched, and the antenna will transfer the harvested RF energy to the IC for triggering of the modulation and back-scattering processes.

(3) $$S11=\Gamma =\frac{z_{chip,tag}-z_{ant,tag}}{z_{chip,tag}+z_{ant,tag}}$$

(4) $$RL(\mathrm{dB})=20\log\left|\Gamma \right|=20\log\left|\frac{z_{chip,tag}-z_{ant,tag}}{z_{chip,tag}+z_{ant,tag}}\right|$$

**Figure 5:**
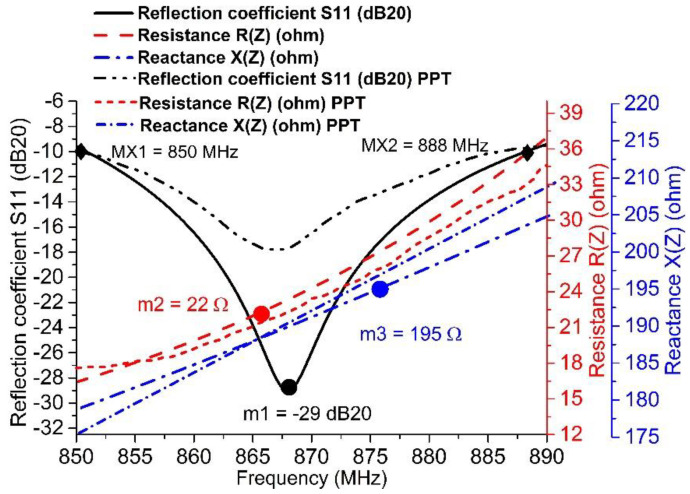
Reflection coefficient measured under −10 dB: m1 is minimum reflection coefficient S11 at *f_c_* = 867 MHz (which is −29 dB), m2 is the resistance (which is 22 Ω), and m3 is the reactance (which is 195 Ω). These markers (m1, m2, m3) exist within the target bandwidth (37 MHz) from MX1 = 851 MHz to MX2 = 888 MHz at S11 = −10 dB and conjugate impedance (22 + *j*195 Ω) of the European UHF RFID band. The tag antenna is achieved w.r.t. the targeted IC impedance (22 − *j*195 Ω) for the proposed tag and previously published tags (PPT).

**Figure 6:**
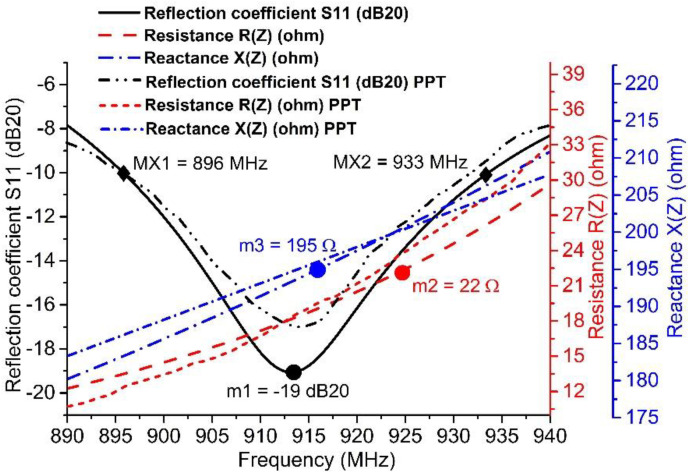
Reflection coefficient measured under −10 dB: m1 is the minimum reflection coefficient S11 at *f_c_* = 913 MHz (which is −19 dB), m2 is the resistance (which is 22 Ω), and m3 is the reactance (which is 195 Ω). These markers (m1, m2, m3) exist within the target bandwidth (37 MHz) from MX1 = 896 MHz to MX2 = 933 MHz at S11 = −10 dB and conjugate impedance (22 + *j*195 Ω) of the North American UHF RFID band. The tag antenna is achieved w.r.t. the targeted IC impedance (22 − *j*195 Ω) for the proposed tag and previously published tags (PPT).

Utilizing Equations (3) and (4) [35], we can calculate the return loss (RL; in dB), which is shown in Figure 5 and Figure 6. This is also called the reflection coefficient (denoted by S11). With the help of the RL curve, the sensitivity and bandwidth of the tag have been measured for the targeted country prerequisites stipulated in Figure 2, as per the UHF RFID standards. In Figure 5, the previously published tags (PPTs) acquired the 40 MHz bandwidth (BW) when the targeted BW to achieve was w.r.t. the EU UHF RFID standard [32]. The PPTs also fulfilled network impedance matching. The PPTs had 22 Ω resistance R(Z) at 867 MHz and 195 Ω reactance X(Z) at 870 MHz w.r.t. the targeted flip-chip package Z_IC_ = 22 − *j*195 Ω, as depicted in Figure 5. However, the proposed tag (PT) achieved 38 MHz BW, 22 Ω resistance R(Z) at 866 MHz, and 195 Ω reactance X(Z) at 873 MHz. In Figure 6, the previously published tags (PPTs) acquired 35 MHz bandwidth (BW) when the targeted BW to achieve was at 26 MHz w.r.t. the NA UHF RFID standard [32]. The PPTs also satisfied network impedance matching. The PPTs had 22 Ω resistance R(Z) at 923 MHz and 195 Ω reactance X(Z) at 911 MHz w.r.t. the targeted flip-chip package Z_IC_ = 22 − *j*195 Ω, as depicted in Figure 6. However, the proposed tag (PT) achieved 38 MHz BW, 22 Ω resistance R(Z) at 924 MHz and 195 Ω reactance X(Z) at 918 MHz. In this way, both the PPTs and the PT obeyed the rules of perfect impedance matching network (PIMT). Due to this characteristic, tag communication is possible in a good manner.

### 2.4. Capacitive Antenna-End Tip-Loading

The introduction of tip-loading at the end of the tag antenna is an additional process for tag antenna size reduction, which is known as capacitive antenna-end tip-loading. Due to radiation, a large number of charges will be accumulated toward the antenna-end surface, expanding the capacitance. Capacitance is inversely proportional to the resonance frequency, which is why when the end tip-loading surface area is enhanced, the resonance frequency is decreased. In this way, the resonance frequency will shift to a lower frequency and the antenna length will be electrically but not practically improved [36]. This process is additionally intended to lessen the antenna’s capacitive reactance. A tip-loaded MAT antenna has better inductance behavior than an antenna that has no tip-loading. Due to the great deal of charges on the antenna-end tip-loading, the bandwidth of the antenna increases at resonance frequency. This does not imply that end tip-loading requires a large surface area for extreme charges, yet it must be present in the antenna structure for a determined bandwidth and for antenna size reduction [13]. Two types of antenna-end tip-loading are presented, one of which is rectangular and other is small-square, as shown in Table 3. The small-square end tip-loading is accomplished by the deduction of five smaller rectangles (2 mm × 1 mm) from the rectangular type.

## 3. Results and Discussions

This section presents the simulation results for the proposed MAT I-RFID UHF tags, in order to facilitate the fabrication of low-profile tags for smart IoT environments in 5G systems. The simulations were performed by implementing the following three realistic scenarios:

- Dipole meandering with MAT (Section 2.1 and Section 2.2);
- Perfect impedance matching (PIM) with the series and shunt stub technique (Section 2.3); and
- Antenna inductive behavior with capacitive end tip-loading (Section 2.4).

The evaluation of the performance of the proposed MAT was quantified in terms of the tag antenna design and simulation detailed in Section 2. To evaluate the impact of the tag on the system performance, the proposed I-RFID tags were placed under the varying humidity conditions, the tag’s read range (RR) was assessed using the Friis equation, and the reader power sensitivity (P_R_) was assessed using the radar equation. Next, we inspected the indoor and outdoor work mechanisms of the proposed tags for IoT in smart X environments. Finally, the performance of the proposed tags was justified on a plethora of platforms with varying characteristics (e.g., bandwidth variation, circuit gain, read range, and reader power sensitivity).

**Figure 7:**
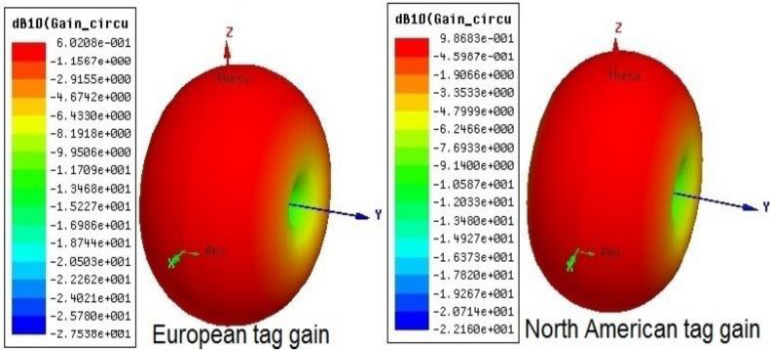
Torus-shaped omnidirectional radiating pattern of antenna gain: for European tag, 0.6 dB (2.75 dBi); for North American tag, 0.98 dB (3.14 dBi).

Figure 5 and Figure 6 outline the simulation outcomes of the proposed UHF RFID tags for the EU and NA standards [32]. Return loss, resistance, and reactance are elaborated in the figures, where S11 is −29 dB and −18.5 dB at 867 MHz and 915 MHz, respectively, while the resistance R(Z) is 22 Ω for both 865 MHz and 924 MHz and the reactance X(Z) is *j*195 Ω for both 873 MHz and 917 MHz. Figure 7 shows the perfect torus-shaped omnidirectional radiating pattern of both antennas’ gains (which were 2.75 dBi and 3.14 dBi, respectively). In comparison with the results in [16], 23.6% to 33.12% improved tag gain was observed.

### 3.1. Meandering Angle Characteristics

**Figure 8:**
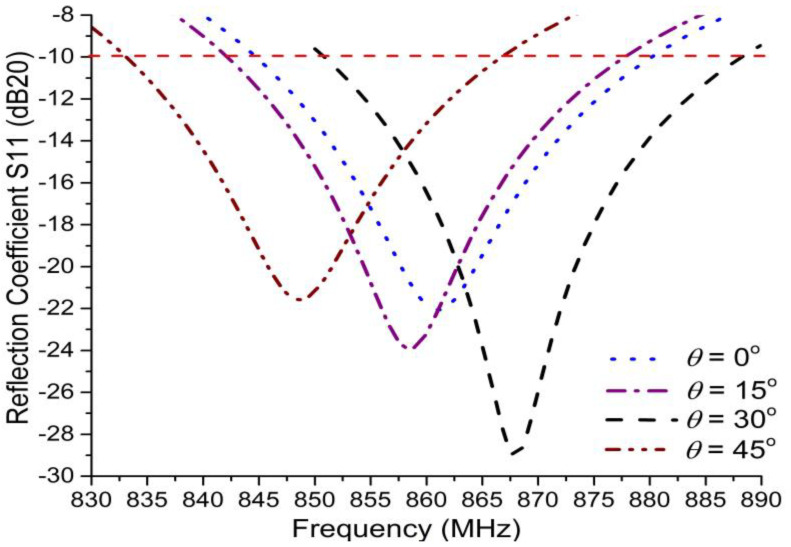
The effect of meandering angle variation on reflection coefficient (S11). With meandering angle variation, the minima of S11 curves fluctuates from −22 to −29 dB, while the tag antenna bandwidth is a little bit reformed (from 33 to 37 MHz). The tag antenna has a minimum reflection coefficient of −29 dB and maximum bandwidth of 37 MHz under −10 dB S11 at *θ* = 30°.

Considering MAT, Table 2 illustrates the behavior of the proposed I-RFID tag antenna, with respect to the S11 curve, circuit gain, and size reduction. At a plane angle (*θ* = 0°), the antenna size was 10.12 cm × 1.22 cm, the minimum reflection coefficient was −22 dB, and the radiated gain was 2.38 dBi. With an elevation angle variation of *θ* = 0°, 15°, 30°, and 45°, the results were adjusted, as depicted in Table 2 and Figure 8. At the angle *θ* = 30°, the tag antenna size was minimized, with lower S11 than with lower angles ones, which led to 10.12 cm × 1.05 cm size and −29 dB S11. The tag antenna gain was 2.75 dBi at *θ* = 30°. Accordingly, the tag is distinguishable at an extreme RR of the relating interrogator/reader, where it has lower power and secures the greatest conceivable bandwidth (37 MHz) at *θ* = 30°. This implies that the MAT affects not only the reflection coefficient (S11) and bandwidth (BW) but also the size and gain of the antenna. The size of the EU tag antenna previously proposed in [16] was 98 mm × 15 mm, while the proposed EU tag had an antenna size of 101.2 mm × 10.5 mm, which was 38.3% smaller in size. The previous NA tag antenna size was 97 mm × 13 mm [16], while the proposed NA tag antenna size was 92.4 mm × 10 mm, which was 36.5% smaller in size.

### 3.2. Series and Shunt Stubs Impedance Matching

For a PIMN, the IMT is important. The considered flip-chip package has a capacitive impedance of Z_IC_ = 22 − *j*195 Ω. Therefore, a typically inductive-behavior antenna should be structured with Z_A_ = 22 + *j*195 Ω impedance. In Figure 4a,b, the proposed antenna attains PIMN using a series and shunt stubs IMT. The conjugate impedance (22 + *j*195 Ω) of European UHF RFID band tag antenna was achieved w.r.t. the targeted IC impedance (22 − *j*195 Ω) for the proposed tag and previously published tags (PPT), as shown in Figure 5 and Figure 6. At this stage, the size of the shunt/short stub is 23.36 mm × 1 mm, while the series/open stub has size 28.8 mm × 1.2 mm [33]. After attaining the PIMN, the harvested electromagnetic power of the tag antenna is shifted to the loaded flip-chip, and its sensitivity is improved. Due to this power sensitivity, data modulation and back-scattering phenomena can be carried out. Along these lines, the IC includes a 32-bit password [28] encrypted data set of object/devices, which will be remotely public with an ideal interrogator or reader within a secure transmission [3].

### 3.3. Capacitive End Tip-Loading Bandwidth Effect

Under a −10 dB reflection coefficient, the antenna bandwidth (BW) is controlled by antenna capacitive end tip-loading. As illustrated in Table 3, if tip-loading obtained the full surface of the 10 mm × 3 mm rectangle, then the BW at S11 = −10 dB is only 28 MHz and the BW at S11 = −15 dB is not estimated. If five smaller rectangles (with 2 mm × 1 mm size) are subtracted from the tip-loading surface area, then an area of 20 mm^2^ will remain. Therefore, the tag antenna BW with small-square end tip-loading is improved, up to 38 MHz at S11 = −10 dB and 20 MHz BW at S11 = −15 dB. This implies that the general return loss is enhanced, and the tag sensitivity is improved.

### 3.4. Dielectric Effect w.r.t. Atmospheric Humidity Level

**Figure 9:**
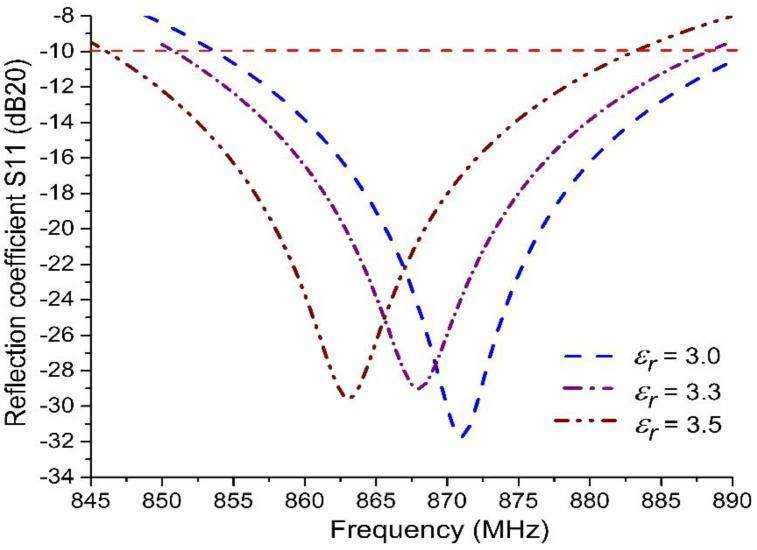
Reflection coefficient results w.r.t. dielectric change (ε_r_) due to humidity-altering effect.

When the humidity level changes with the variation of environmental temperature, the dielectric (*ε_r_*) of the substrate (Paper Korsnäs) will be affected. Figure 9 shows that when the substrate dielectric becomes *ε_r_* = 3.0, the reflection coefficient curve will be shifted 5 MHz to a higher frequency; furthermore, it will shift 5 MHz to a lower frequency from the targeted center frequency (*f_c_*) when the substrate dielectric becomes *ε_r_* = 3.5. In the above scenario, the proposed MAT tag holds its characteristics for the EU UHF band standard with humidity level inflation over the dielectric (*ε_r_*) from 3.0 to 3.5. Similarly, the MAT tag will also bear all environmental circumstances for the NA UHF standard. Hence, the substrate materials are not influenced by the general results of proposed tag antenna whose permittivity (*ε_r_*) ranges from 3.0 to 3.5. It implies that such tag substrate materials can be utilized well, and its performance should not be affected during summer and winter spells. In other words, it is reliable throughout the year.

### 3.5. Tag Read Range (RR) w.r.t. Reader’s EIRP

**Figure 10:**
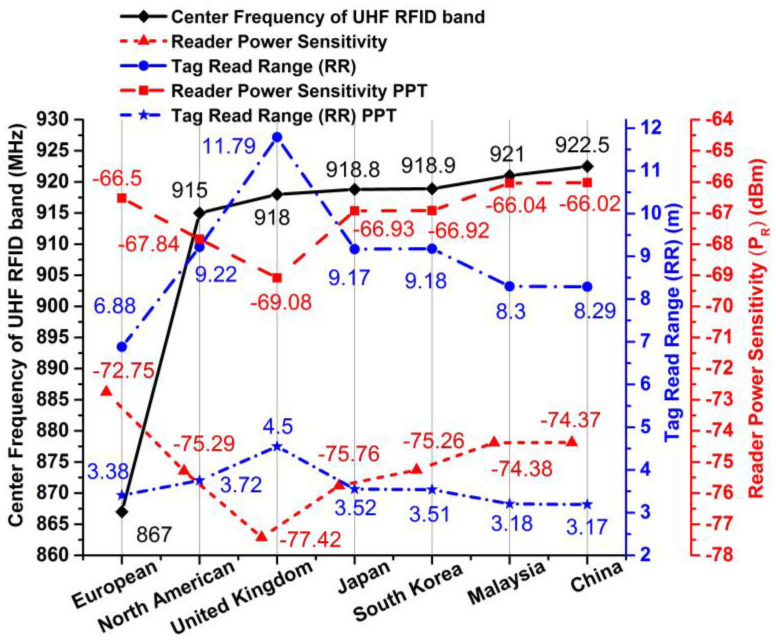
Read range (RR) and reader power sensitivity (P_R_) for UHF RFID band countries w.r.t. center frequency (*f_c_*) for proposed tags and previously published tags (PPT).

For massive industrial usage, the read range (RR) is another debatable boundary of IoT in smart 5G systems. In this way, it cannot be disregarded when an RFID tag is examined and the Friis conditions assume a significant job to uncover the RR (*d_max,read_*) [10,26,31,37,38,39,40,41,42,43,44], as shown in Equation (5):

(5) $$d_{\max,read}=\frac{c}{4\pi f}\sqrt{\frac{EIRP_{reader}.G_{ant,tag}.\tau }{P_{chip,tag}}}$$

(6) $$\tau =\frac{4R_{chip}.R_{ant}}{{\left|z_{chip}+z_{ant}\right|}^{2}}\leq 1$$

where *c* (speed of light) = 3 × 10^8^ m/s, *f* is the targeted frequency, the *EIRP* (effective isotropic radiated power) is set as a standard by state spectrum regulations—for example, *EIRP* = 3.28 W for the EU UHF standard and *EIRP* = 4 W for the NA UHF standard—*τ* is the transmission loss, and *P_chip,tag_* = 0.0316 × 10^−3^ W (−15 dBm). *τ* should be unity or less than unity, and it is calculated using the resistances and impedances of the flip-chip package and the manufactured antenna. In the given case, the extreme RRs of reader are 6.88 and 9.22 m for the proposed tags, where the MAT tags can accept the threshold power of the flip-chip. These RRs constitute improvements of 50.9% and 59.6% over the formerly published case in [16] within EU and NA bands [32], respectively. By overhead estimations, the interrogator/reader P_R_ is considered, in which the tags are in dynamic mode and the RR is calculated at the targeted frequencies for each country. These are shown in Figure 10 for the previously published tags (PPTs) and proposed tags (PTs), correspondingly. Both tags have dominant RR in the IoT environment w.r.t. the respective UHF band rules and regulations [32], but the PTs outperform the PPTs in all country tiers. The PT achieved the maximum RR (11.79 m) for the U.K. band, while the PPT had an RR of only 4.5 m. The PPT attained the minimum RR (3.17 m) for the Chinese band, while the PT outperformed in the European band with an RR of 6.88 m, constituting a 50.9% improvement for that case. The PT RR was 5.5 m higher than that of the PPT in the North American case. In all scenarios, the PTs performed better than the PPTs, as portrayed in Figure 10.

### 3.6. Reader Power Sensitivity (P_R_)

Another negotiable constraint is the power sensitivity of the interrogator/reader. This is measured by the radar equation and the interrogator simply reading the MAT tag during back-scattering after data modulation. The radar equation is given as [10]:

(7) $$P_{R}=\frac{\sigma .EIRP_{reader}.G_{ant,tag}}{4\pi }{\left[\frac{c}{4\pi f.d_{\max,read}^{2}}\right]}^{2}$$

where *P_R_* is the reader power sensitivity and *σ* is the radar cross-section, which is 0.001 m^2^. Then, by utilizing Equation (7), we calculated the *P_R_* = −72.75 dBm for the European (EU) band and *P_R_* = −75.29 dBm for the North American (NA) band. The *P_R_* value for each UHF RFID band country is likewise categorized in Figure 10 for the previously published tags (PPTs) and proposed tags (PT), separately. The reader power sensitivity (P_R_) ranged from −69.08 dBm to −66.5 dBm for the PPTs and from −77.42 dBm to −72.75 dBm for the PTs. Put simply, we can say that a reader would require 1.58 × 10^−4^ mW power to communicate with the PPTs; meanwhile, for the detection of PTs, they need a minimum 3.16 × 10^−5^ mW of power. Through comparison of these two scenarios, the PTs performed better than the PPTs. In the smart IoT environment, all proposed tags have a radar coverage area (RR) of 6.88 m and 9.22 m for EU and NA bands, respectively. Consequently, the reader sensitivity should be less than the *P_R_* value, in order to receive data from the tag at its corresponding RR (*d_max,read_*). This implies that MAT plays a massive role in all basic project prerequisite characteristics of the tag. 

### 3.7. Experiment and Printing Setup

**Figure 11:**
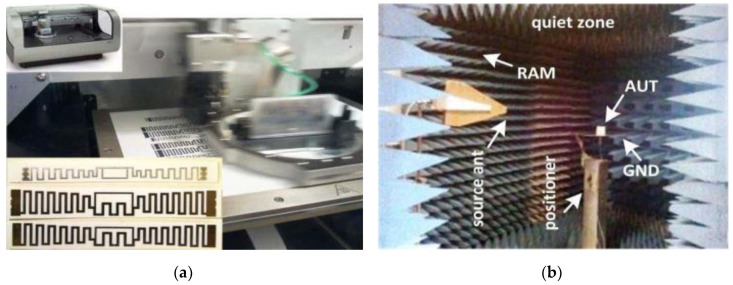
(**a**) Inkjet printing setup and paper substrate printed antennas; (**b**) Experimental setup in an anechoic chamber [16].

During shipment and freightage, it is normal that goods get nicked. Therefore, to solve this issue, I-RFID tags were produced utilizing flexo- and screen-printing with Asahi conductive paste. Exceptional mechanical accomplishment (i.e., antenna conductive efficiency) was noticed by us after inkjet printing (see Figure 11a) [16]. Inside the anechoic chamber shown in Figure 11b, the near-field execution of tags was done using Imping’s UHF RFID reader kit. The maximum radiation structure (*D*) of Imping’s kit was 0.3 m. The far-field distance within the anechoic chamber is 0.56 m, as calculated by *2D^2^/λ* at 915 MHz (*λ* = 0.33 m). The tags proposed in [16] exhibited flawless readability within the anechoic chamber at *2D^2^/λ*.

By utilizing a vector network analyzer (MS2026b, Anritsu), impedance assessments were carried out using the short-open-load (SOL) standard calibration method [16]. Figure 5 and Figure 6 show that the resistance and reactance of the proposed tags were achieved within the target bandwidth. For the EU UHF RFID band, 24 Ω resistance and 191 Ω reactance were measured at the target frequency of 867 MHz; similarly, 20 Ω resistance and 191 Ω reactance were measured at the target frequency of 915 MHz for the NA UHF RFID band. These values were very close to the targeted flip-chip package NXP G2XM (22 − *j*195 Ω). Thus, good agreement was found between the proposed series and shunt stubs of the tag and the flip-chip package. 

As the proposed tag substrate was the paper-based material Korsnäs, the permittivity variation (due to atmospheric humidity levels) behavior is important in determining the final tag performance. Figure 9 shows that only 5 MHz center frequency shifting was observed under varying humidity, which means that the tag will cover the required targeted bandwidth (3 MHz for the EU UHF RFID standard and 26 MHz for the NA UHF RFID standard) in all-weather circumstances w.r.t. humidity level variations.

For the low-profile I-RFID tag design, Asahi paste inkjet-printed tags have larger return loss values compared to the return loss of copper tags, and the antenna radiation pattern was measured in an anechoic chamber setup that mimics infinite free-space, as depicted in Figure 11b [16]. The final omnidirectional radiation pattern of the proposed antenna gain for both UHF RFID tags is shown in Figure 7, which was 2.75 and 3.14 dBi for EU and NA UHF RFID standards, respectively. 

**Figure 12:**
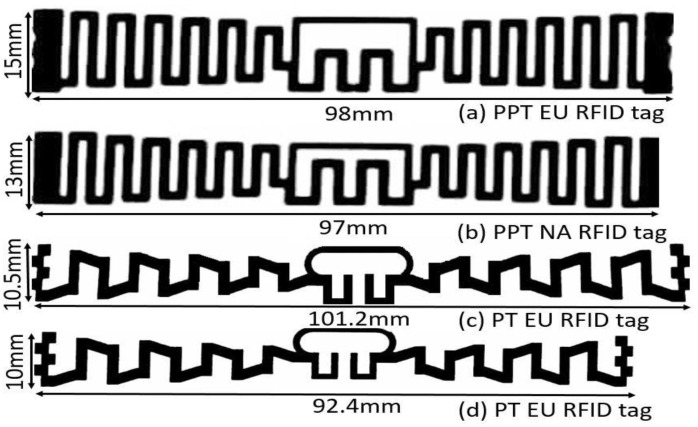
Prototypes of the antennas of previously published tags (PPT) and proposed tags (PT): (**a**) PPT EU UHF RFID tag (98 × 15 × 0.39 mm^3^); (**b**) PPT NA UHF RFID tag (97 x 13 × 0.39 mm^3^); (**c**) PT EU UHF RFID tag (101.2 × 10.5 × 0.39 mm^3^); and (**d**) PT EU UHF RFID tag (92.4 × 10 × 0.39 mm^3^).

Antenna prototypes of previously published tags (PPTs) and proposed tags (PTs) are depicted in Figure 12 (a and b) and (c and d), respectively, for UHF I-RFID. Low-profile and cost-effective design is key for the both manufacturers and users in smart environments. The dimensions of the previously proposed European tag antenna were 98 mm × 15 mm, while the proposed European tag antenna had dimensions of 101.2 mm × 10.5 mm, which was 38.3% smaller in size and dimension. The previous North American tag antenna [16] was 97 mm × 13 mm, while the proposed NA tag antenna dimension was 92.4 mm × 10 mm, which was 36.5% smaller in size with respect to the previously published tag. Thus, MAT reduced the complexity and size of the antennas (up to 37%) at the angle of *θ* = 30°.

### 3.8. Comparison between Proposed Tags (PT) and Previous Published Tags (PPT)

Table 4 compares the proposed tags (PT) with the previously published tags (PPT), in terms of their structure, for several types of applications based on tag dimension, volume, effective gain, bandwidth, and read range. The PTs had minimal three-dimensional properties and volume (EU 101.2 mm × 10.5 mm × 0.39 mm = 414.414 mm^3^; NA 92.4 mm × 10 mm × 0.39 mm = 360.36 mm^3^), compared to the others. The PPTs were fabricated with radiating materials (e.g., aluminum foil, copper, metals) and substrate materials (e.g., FR4, polycarbonate, polyethylene terephthalate (PET), alumina/Al_2_O_3_). These materials are not suitable for low-profile tag production. The PTs also have a pliable and flexible feature, which makes their usage more convenient. 

The PTs are intended to be used in IoT applications as green tags, as they are easily disposable without bad environmental effects. The effective gain of the PTs is maximal, compared to the PPTs, leading to long-range communication with a perfect impedance matching network. In terms of tag sensitivity, the PPTs of [17,18,22,23] were less sensitive, as the bandwidth was estimated under the S11 of −10 dB, where they had less BW. The incredible RR of our tags (6.88 and 9.22 m for EU and NA UHF RFID bands, respectively) were greater than the other PPT cases in Table 4. The proposed tags satisfy the requirements of low-profile design, considering their size, use of materials with high effective gain, incredible read range, and sensitivity.

### 3.9. I-RFID Indoor and Outdoor Work Mechanism for IoT in Smart X Environments

I-RFID is generally considered a prerequisite for IoT in smart X environments. I-RFID technologies play a dynamic role in challenging IoT environments of smart cities, such as securing massive logistics control at seaports and aerodromes. At present, more goods are passing through the world’s shipping terminals than ever before. It is difficult to prevent explosions when using the large, static detectors that are basically utilized for detecting explosive materials. Passive I-RFID tags, which work as remote, battery-free explosive detectors, can be mounted practically everywhere in challenging IoT environments. On the other hand, product misplacement and chances of evasion have also been increasing with the passage of time. To overcome such issues, I-RFID tags can play an enormous role for goods management in a secure manner in smart cities.

In retail environments, technological change has been happening fast. For this reason, many retailers have been shifting their products toward the use of I-RFID in order to improve the unprecedented level of inventory accuracy provided by a smart-friendly IoT environment. Within such a smart environment, consumer satisfaction can be upgraded, which is basic tenet of product marketing. Some stores are currently in the process of introducing frameworks that permit personal telephones to receive messages when an individual enters the store. Ongoing offers are sent to the phone, informing the individual of offers accessible in that store and enabling them to have a more brilliant shopping experience in the IoT environment. We know that everything is wrapped or packed with a plastic cover or cardboard box for retailing and shipping. For this reason, the proposed tag’s performance has been optimized for such platforms (e.g., plastic, paper, and glass).

For example, the UHF RFID integrated reader SL130 is manufactured by the “Strong link” [30]. It is especially designed for vehicle management, electronic toll collection (ETC), personnel access control, anti-counterfeiting, logistics monitoring, and product auto management systems. It works for both European and North American UHF bands within a tag reading distance of about 8 m. Its output power is 20 to 30 dBm. The triggering on power of the proposed NA tag is −75.29 dBm. This means that more than 3 million tags can be activated with the 20 dBm power of the reader (SL130). The reading speed of the SL130 is 32 bits/6 ms.

**Figure 13:**
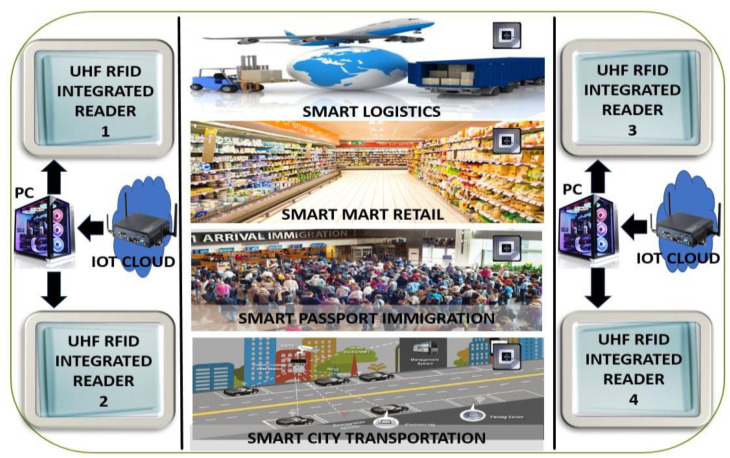
I-RFID indoor and outdoor work mechanism for IoT in smart X environments (e.g., smart logistics, smart market retail, smart passport immigration clearance, and smart city transportation).

The proposed tag IC memory is 512 bits, which can be read by the SL130 within 96 ms. In one second, the SL130 can easily read approximately 10 tags and, so, 600 tags can be read by the SL130 within 60 s. If four concatenated readers are placed in any smart environment, as depicted in Figure 13, then 2400 of the proposed tags can be read by them per minute. This is a milestone for IoT in smart X environments, facilitating supply chain management through smart logistics, product retailing at smart marts, immigration clearance by smart passports, and transportation and surveillance in smart cities.

### 3.10. Tag Performance on Mounting Platforms

**Figure 14:**
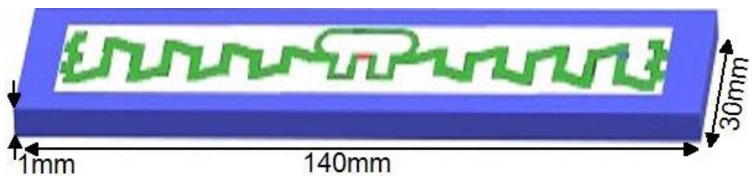
Simulation model for all platforms (140 mm × 30 mm × 1mm) w.r.t. the RFID tag.

A simulation model for the platforms is shown in Figure 14, and the platform’s dielectric properties (i.e., permittivity and tangent loss) are given in Table 5 [31]. Each platform’s dimension is 140 mm × 30 mm × 1 mm, with respect to the designed UHF RFID tag. Table 6 elaborates the tag performance on several mounting platforms (i.e., plastic, paper, glass, and water). The proposed North American UHF RFID tag’s bandwidth at center frequency (*f_c_* = 915 MHz) is 37 MHz. There was no variation in bandwidth on plastic and paper platforms, while glass and water platforms showed 3 MHz and 7 MHz detection in the proposed tag’s bandwidth, which is enough to cover the specified required bandwidth.

On the other hand, center frequency (*f_c_*) variation could not be ignored, as the RFID UHF bandwidth limitation is 860–960 MHz. The *f_c_* values of plastic and paper platforms still fell within the required band, but those of glass and water were outside the RFID UHF bandwidth specified in Table 6. By parametric optimization, the *f_c_* values of plastic, paper, and glass can be shifted to the specified UHF RFID band for the relative country.

**Figure 15:**
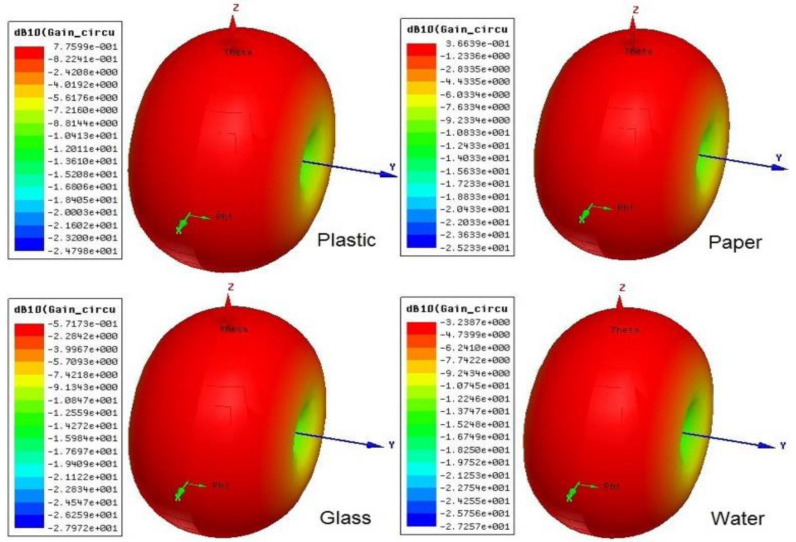
Torus-shaped omnidirectional radiating pattern of antenna gain for North American UHF RFID tag on several mounting platforms: 0.77 dB (2.92 dBi) for plastic; 0.36 dB (2.51 dBi) for paper; −0.5 dB (1.58 dBi) for glass; and −3.2 dB (−1.05 dBi) for water.

**Figure 16:**
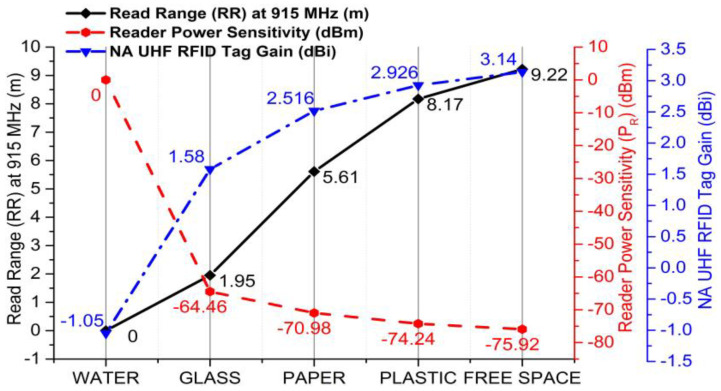
Tag gain read range (RR) and reader power sensitivity (*P_R_*) of NA UHF RFID tag on mounting platforms and in free space.

The circuit gain and read ranges of tags are likewise significant parameters, which are depicted in Table 6 for each platform. The circuit gains of plastic, paper, glass, and water were 2.926, 2.516, 1.58, and −1.05 dBi, respectively; their 3D polar plots are shown in Figure 15. By utilizing Equations (5) and (6), we calculated the decent read ranges of the NA UHF RFID tags, which were 8.17 and 5.61 m on plastic and paper platforms, respectively. On the glass platform, the tag read range was 1.95 m, which is significant. The tag had no grip on the water platform w.r.t. circuit gain and read range, which are part of Table 6. The read ranges of the NA UHF RFID tag w.r.t. several platforms and in free space are portrayed in Figure 16.

The reader/interrogator sensitivity of the tag on each platform is depicted in Table 6 by using Equation (7), where the reader requires a minimum of −74.24 dBm of power to detect the plastic platform tag at a distance of 8.17 m, while for paper and glass, the reader sensitivity is −70.98 and −64.46 dBm, respectively. The line graph in Figure 16 elaborates the tag gain, RR, and reader power sensitivity statistics of the NA tag in free space and on mounting platforms. Here, the line graph indicates the relationships among the tag gain, read range, and reader power sensitivity. Whenever the tag gain is diminished, the read range is straightforwardly affected and the reader power sensitivity is enhanced, respectively. For the paper platform, the gain of the tag was 2.516 dBi and the read range was 5.61 m; with respect to these, the reader power sensitivity was −70.98 dBm.

## 4. Conclusions 

In this paper, minimized Passive I-RFID UHF meander-line antennas were designed using the meandering angle technique (MAT). The resultant gain was 2.75 and 3.14 dBi for European (EU) and North American (NA) standards, respectively. The I-RFID tag size was reduced by 36% to 38% w.r.t. previously published cases, the gain of the proposed tag was improved by 23.6% to 33.12%, and its read range was enhanced by 50.9% and 59.6% for EU and NA UHF bands, respectively. Our results show that the tag antenna was also optimized with respect to humidity, allowing its use in IoT systems in smart X environments in all weather conditions. Small tags were utilized for UHF I-RFID applications, where the applicable RR was 6.88 m (EU) and 9.22 m (NA) and the maximum bandwidth was 38 MHz at S11 = −10 dB for the global UHF standard (860–960 MHz). For IoT challenges in smart X environments, the proposed tag retains impressive properties when mounted on plastic, paper, and glass platforms. The proposed tag has powerful and graspable outcomes; for example, for locating books in smart computerized libraries, for supply chain management in smart marts, to secure massive logistics control for smart seaports and aerodromes, for smart highways and vehicle identification, for smart passports to enhance immigration clearance in airports, and for surveillance in smart cities using 5G systems. Supplementary future developments are to optimize the design in order to approach the elastic bandwidth and further miniaturize the design.

## Figures and Tables

**Table 1:** Geometric boundaries of UHF I-RFID tag antennas.

**Values**	***A***	***B***	***C***	***D***	***E***	***F***	***G***
(mm)	101.2	10.5	1	1.2	1.6	3	2
**Values**	***H***	***I***	***J***	***K***	***L***	***θ***	
(mm)	0.015	92.4	10	1	1	30°	

**Table 2:** Meandering angle characteristics and parameters for both European (EU) and North American (NA) UHF band standards.

Angle (Degree)	EU/NA UHF Band	S11 (dB)	Bandwidth (BW) (MHz) at −10 dB	Compact Size (cm^2^)	Gain (dBi)
*θ* = 0°	EU	−22	36	10.12 cm × 1.22 cm	2.38
*θ* = 15°	EU	−23.9	35	10.12 cm × 1.10 cm	2.28
*θ* = 30°	EU	−29	37	10.12 cm × 1.05 cm	2.75
*θ* = 45°	EU	−21	33	10.12 cm × 1.06 cm	1.75
*θ* = 30°	NA	−18.5	37	9.24 cm × 1.0 cm	3.14

**Table 3:** Qualities and boundaries of tag antenna-end tip-loading.

Name	Shape	Area	Bandwidth at (S11 = −10 dB)	Bandwidth at (S11 = −15 dB)
Rectangular	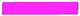	30 mm^2^	27 MHz	n/a
Small-Square	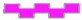	20 mm^2^	37 MHz	20 MHz

**Table 4:** Comparison of proposed tags with miscellaneous previously proposed tags. (n/a), not available. Required bandwidth (at S11 = −10 dB) is 3 MHz and 26 MHz for European (EU) and North American (NA) UHF RFID bands [32], respectively.

Ref.	EU/ NA	Tag Dimension (mm^3^)	Gain (dBi)	Bandwidth (BW) (MHz) at −10 dB	Read Range (RR) (m)
[16]	EU	98 × 15 × 0.39	2.1	51	3.38
	NA	97 × 13 × 0.39			3.72
[17]	EU	49.14 × 17.9 × 0.5	1.7	8	5.95
[18]	NA	140 × 60 × 10	n/a	0	7.5
[20]	NA	93 × 23 × 8	1.5	60	5
[21]	EU	92 × 33.54 × 1.38	1	22	4
	NA	87.8 × 31 × 1.31	1.5	28	5
[22]	EU	85 × 40 × 3.2	n/a	8	~7.5 (−15 dBm)
[23]	EU	23 × 23 × 1	n/a	n/a	1
Ours	EU	101.2 × 10.5 × 0.39	2.75	37	6.88
	NA	92.4 × 10 × 0.39	3.1	37	9.22

**Table 5:** Dielectric properties of the mounting platforms [31].

Platforms	Permittivity (*ε_r_*)	Tangent Loss (*δ*)
Plastic	3	3
Paper	3.2	0.07
Glass	4.82	0.0054
Water	77.3	0.147

**Table 6:** North American UHF RFID tag performance on several platforms and in free space.

Platforms	*f_c_* Variation	Bandwidth Variation	Circuit Gain (dBi)	Read Range (m)	Sensitivity (dBm)
Free Space	0	0	3.14	9.22	−75.92
Plastic	−45	0	2.926	8.17	−74.24
Paper	−47	0	2.516	5.61	−70.98
Glass	−78	−3	1.58	1.95	−64.46
Water	+80	−7	−1.05	0	0
